# Supplementary material for: Study protocol for cholera vaccination as a model to measure the inflammatory response in the gut: A case of modulation with a Lactobacillus plantarum K8 lysate
Source: PLoS One. 2023 Feb 21;18(2):e0281817. doi: 10.1371/journal.pone.0281817 (PMC9942990; doi:10.1371/journal.pone.0281817)
Supplement: S2 File — (PDF) [file pone.0281817.s002.pdf]

Cholera vaccination as a model to measure the inflammatory response in the gut: A case of modulation with a *Lactobacillus plantarum* K8 lysate

Protocol No.: K8_SP

Version No.: 1.7

Version Date: 2019. 01. 29.

Signature Page

I will not initiate this study without approval of the appropriate Institutional Review Board and I understand that any changes in the protocol must be approved in writing by the sponsor and the Institutional Review Board before they can be implemented, except where necessary to eliminate immediate hazards to the subjects.

By my signature below, I attest that I have read, understood, and agree to abide by all conditions, instructions, and restrictions contained in this protocol.

________________________	________________________	________________________
Principal Investigator	Co-investigator (Chief investigator)	Sponsor
________________________	________________________	________________________
Title	Title	Title
________________________	________________________	________________________
Date	Date	Date

1. Background

An effectively functioning immune system is crucial for maintaining physiological integrity, and thus health. The immune system provides defense against infections caused by pathogenic microorganisms. European Food Safety Authority (EFSA) considers that maintaining a normal immune function is a beneficial physiological effect. Defense against pathogens comprises different mechanisms which act in concert to protect against infection. The presence of pathogenic microorganisms may cause infections at various sites of the body, and the defense against pathogens at a specific site of the body is considered a beneficial physiological effect. The capacity for defense against pathogens in the gastro-intestinal tract may be a good clinical trial model for proving gastro-intestinal health. Additionally, immune response to vaccination is an acceptable outcome to substantiate a beneficial physiological effect on the immune system.

Inflammatory responsiveness or resilience to challenges may provide a more sensitive and meaningful indication of inflammatory state in the general population than the assessment of markers during the steady state and potentially the early response to a vaccine seem to be best standardized, most relevant and most feasible challenges for application in nutrition studies. Dietary modulation of the response to vaccination seems to be a good model for stimulating gastrointestinal inflammation and increasing gut permeability.

The oral cholera vaccine (Dukoral®) consists of 1.25× 10^{11} heat- and formalin-killed *Vibrio cholerae* bacteria and 1 mg recombinant cholera toxin B-subunit (rCTB). CTB is a well-characterized nontoxic yet potent mucosal immunogen, partly because of its high-affinity binding to the receptor GM1 ganglioside. In order to induce a strong local immune response after vaccination, a whole-cell preparation or a protein with mucosa-binding properties is required. The vaccine is given in a sodium bicarbonate solution. It is hypothesized that the binding of the

CTB induces an inflammatory reaction. How strong or how extensive the inflammatory reaction is, has not been investigated yet. In a recent study in which NIZO food research has used the cholera vaccine, calprotectin will be measured in fecal samples - these results will give a first idea of the inflammatory component of the vaccination (www.clinicaltrials.gov NCT02238548). In the present study, we can check the appropriate time point for the peak response of calprotectin, also.

Lactobacillus plantarum is a probiotic listed in the Korean Health Functional Food Code, and is a representative probiotic that appears in the fermenting season among the lactic acid bacteria contained in Kimchi which is a fermented food of Korea. SEMPIO Foods Company developed nano-sized *Lactobacillus plantarum* K8 lysate by separating *Lactobacillus plantarum* K8 from Kimchi and disrupting using a physical method.

The production of pro-inflammatory cytokines by the mucosa and by immune cells may increase immune cell infiltration. A number of studies have reported that the administration of live or killed bacteria of the *Lb. plantarum* genus decreases colonic pro-inflammatory cytokines and/or increases anti-inflammatory cytokines (Geier *et al*., 2007, Mileti *et al.*, 2009, Chen *et al*., 2012, Fujiki *et al*., 2012, Zhou *et al*., 2012). Although probiotics are generally defined as live microorganisms, published evidence suggests that lysates or components isolated from probiotics function similarly to probiotics (Kataria *et al*., 2009, Adams, 2010, Zakostelska *et al*., 2011). Specifically, recent studies have reported that lipoteichoic acid (LTA), which is a cell wall component, obtained from *Lb. plantarum* K8 induces lipopolysaccharide (LPS) tolerance and reduces the excessive production of pro-inflammatory cytokines and nitric oxide in the macrophage or monocytic cell lines (Ryu *et al*., 2009, Kang *et al*., 2011, Kim *et al*., 2011). Therefore, LTA from *Lactobacillus plantarum* K8 may control the homeostasis of intestinal inflammation. According to our previous study, *Lb. plantarum* K8 *lysate* was found to protect against damage to the histological mucosa and disruption of the balance of the immune system induced by DSS colitis (Ahn et al., 2014). *Lb. plantarum* K8 *lysate* decreased neutrophil infiltration

of the colonic mucosa and inhibited the production of pro-inflammatory cytokines, including TNF-α and IL-6, and IL-1β mRNA levels. Furthermore, DSS-induced colitis symptoms, such as weight loss, diarrhea, fecal bleeding, and shortened colon length, were reduced. In particular, a high dose of *Lb. plantarum* K8 lysate may mediate the TLR-2 signaling that is associated with tight junctions. Therefore, we suggest that the improvement in colonic immune function in the high-dose *Lb. plantarum* K8 lysate (1×10^{10} CFU/day)-administered group may result from TLR-2-mediated signaling and/or immune system regulation via the induction of LPS tolerance stimulated by LTA as a low-grade inflammation.

In this study, we aim to develop a new human model that can be used to study gastrointestinal inflammation. Because of the immunogenicity of the cholera vaccine which targets the gut, we would like to provide support for our concept that, in addition to inducing a protective immune response, this vaccine may also be used as a safe approach to induce a mild gastrointestinal inflammatory response in healthy human subjects, as a challenge model for dietary intervention studies. To study whether this inflammatory response or the vaccination response can be modulated by an active ingredient, we will use *Lactobacillus plantarum* K8 lysate as intervention.

2. Objectives

The primary objective of this exploratory study is to investigate whether cholera vaccination can be used as a model to induce a mild inflammatory response in gut.

The secondary objective is to study the effect of *Lactobacillus plantarum* K8 lysate on the inflammatory response or on the immune response as induced by oral cholera vaccination.

3. Hypotheses

3.1 Hypothesis 1

The oral cholera vaccination will induce a significant increase in the gut inflammatory response in the gastrointestinal tract.

3.2 Hypothesis 2

Lactobacillus plantarum K8 lysate will significantly improve the inflammatory response or support the immune response induced by oral cholera vaccination in healthy subjects as compared to placebo.

4. Overview of Study Design

This study will focus on gastrointestinal inflammation. The recruitment goal will be 46 subjects, who will be equally and randomly assigned to active treatment or placebo groups. Subjects will give their signed informed consent, provide a blood sample and submit to a brief medical exam to determine study eligibility. Subjects who are eligible will be given instruction on diet control, as well as fecal sample collection methods. At day 0, subjects provide blood sample and receive a two-week supply of study product in blinded, randomized fashion using a list provided by the sponsor. After collecting fecal sample on day 0 (allowed up to day 1 when it is difficult to collect fecal sample on day 0; described as "~day 1" from below), subjects start to intake study product. Subjects will return unused study product, and receive additional study product on day 15 and 29. On day 15 and 29, they will be given the oral cholera vaccine. Subjects should visit the study site on day 0, 15, 16, 17, 29, 31 and 43, and provide blood. Fecal samples should be collected on day 0, 14, 16, 17, 28, 30, 31 and 42.

	Intervention													
**Study day**	**0**	**1**	**14**	**15**	**16**	**17**	**19~27**	**28**	**29**	**30**	**31**	**33~41**	**42**	**43**
**Window period(day)**														**+3**
**Visit time code**	**V1**			**V2**	**V3**	**V4**			**V5**		**V6**			**V7**
Informed consent														
Blood sampling				*					*					
In/Exclusion criteria review														
Randomization														
Vaccination														
Feces sampling		#												#
Sending fecal sample														
Adverse event monitoring														
Questionnaire **														
Dietary guidelines														
Probiotics/placebo														
- Distribution														
- Supplementation														
- Return														

* Collect blood before providing the vaccine!

** Written or online questionnaire using google survey

Choice of timepoints:

1. The vaccination timepoints are fixed on day 15 and day 29.

2. Fecal samples: inflammation may also be reflected in the feces. From previous studies using an *E. coli* challenge, we know that Calprotectin level in feces show a peak on day 2 after challenge. Because the kinetics in the oral cholera vaccination may be similar, fecal samples should be collected on day 17 and 31 (2 days after vaccination). Fecal samples on day 16 and 30 are also collected. Because of natural variation, it is best to have 2 baseline samples. Therefore, we propose to collect fecal samples on day 0 and on day 14 (before vaccination). In order to analyze whether values go back to baseline values, also a sample on day 42 is of value.
   # Fecal sampling would be allowed when it is difficult to collect fecal sample on day 0 and 42.
   ※ If subject cannot collect fecal sample on day 0 or day 1, they would collect fecal sample as soon as possible and start to intake study product after the day of collecting fecal sample.

3. Blood samples: for markers related to inflammation, it is advised to collect samples at the same days as fecal samples. We know from a previous oral cholera vaccination study that the maximum vaccination antibody response is observed 28 days after the first vaccination dose. Therefore, day 43 is an important timepoint for blood collection.

5. Personnel

Role	Name	Affiliation
Principal Investigator	*****	**********<br>Tel: **********, E-mail: **********
Co-investigator (Chief investigator)	*****	**********<br>Tel: **********, E-mail: **********
Co-investigator	*****	**********<br>Tel: **********, E-mail: **********
Co-investigator	*****	**********<br>Tel: **********, E-mail: **********
Co-investigator	*****	**********<br>Tel: **********, E-mail: **********
Research Coordinator	*****	**********<br>Tel: **********, E-mail: **********
Research Coordinator	*****	**********<br>Tel: **********, E-mail: **********
Monitor	*****	**********<br>Tel: **********, E-mail: **********

6. Treatments

All subjects will receive the registered oral cholera vaccine Dukoral® according to the standard vaccination scheme (2 doses, 14 days apart). There will be two treatments groups, one receiving *Lactobacillus plantarum* K8 lysate, and the other receiving placebo. Both products will be coated with an identical color and packaged into bottles. A daily serving consists of two chewable tablets, to be taken as one tablet twice a day.

The composition of the test product and the placebo product is specified in the table below.

(%)

Ingredient	Test product	Placebo product
Lactobacillus plantarum K8 lysate	11.77	-
Refined glucose	86.23	86.23
Lactose	-	11.75
Gardenia yellow	-	0.02
Magnesium stearate	1.50	1.50
Mint flavor	0.50	0.50
Total	100.00	100.00

7. Power Analysis

Since cholera vaccination has not been used before as a model to induce low-grade inflammation, and therefore is a new model, the study will have an explorative character. It is not known how strong the inflammatory signal will be. Therefore, a large panel of biomarkers will be analyzed, of which the data can be integrated for multivariate analysis, which will increase the power of the study. Because it is known that the vaccination will induce an antibody response, the immune response to the vaccination will be included as outcome.

In order to estimate the number of subjects needed for the analysis of the inflammatory response, sample size calculation and power analysis is based on the fecal calprotectin, with additional support from calculation based on the other outcome parameter.

The required sample size was calculated for the calprotectin level in feces. The hypothesis is that the vaccination will induce a low-grade inflammatory response, resulting in an increase in the level of fecal calprotectin as compared to baseline. In diagnostics, fecal calprotectin levels above 50 μg/g feces is considered to be an increased level. From previous studies performed at NIZO food research, we know that the calprotectin level (mean $\pm$ SD) in healthy individuals is 15 $\pm$ 20 μg/g feces. An increase to a mean level of 50 μg/g is considered to be a relevant effect. Based on two-sided statistical testing for paired data, $\alpha=0.05$ (chance on type-I error) and $\beta=0.10$ (chance on type-II error), it was calculated using the software package Statistica (2015) that 19 subjects per group are needed for this outcome ($\mu 1=15$; $\mu 2=50$; SD1=20; SD2=40; $\alpha=0.05$; $\beta=0.10$).

For outcome parameters of systemic inflammation markers, the required sample size was also calculated. This was based on data from literature, showing an increase in systemic inflammatory markers in low grade inflammation associated with obesity. We expect that the level of inflammation that is induced by cholera vaccination in the placebo will be in the same order of magnitude.

For C-reactive protein (CRP), it was shown that the plasma level in low-grade inflammation is 2.54 $\pm$ 2.07 mg/L (mean $\pm$ SD), as compared to 1.01 $\pm$ 0.88 mg/L in normal individuals (Brignardello et al, 2010). Assuming that cholera vaccination will induce a similar extent of CRP increase as compared to baseline in the placebo group, based on two-sided statistical testing for paired data, $\alpha=0.05$ (chance on type-I error) and $\beta=0.20$ (chance on type-II error), it was calculated that 18 subjects per group are needed for this outcome ($\mu 1=1.01$; $\mu 2=2.54$; SD1=0.88; SD2=2.07; $\alpha=0.05$; $\beta=0.20$).

Therefore, we aim to enroll 23 subjects in each group assuming 20% drop-out rate.

7.1 Inclusion Criteria

- Signed informed consent
- Age 20-50 yr
- Male
- Healthy bowel habit
- Availability of internet connection
- Smart phone user

7.2 Exclusion Criteria

- Previous cholera vaccination.
- Other vaccination in the past 1 month.
- Acute gastroenteritis in the past 2 months.
- Use of antibiotics in the past 2 months.
- Hypersensitivity to the vaccine, to formaldehyde, to any of the excipients (sodium salts) or to probiotics.
- Disease of GI tract (constipation, diarrhea, IBD, etc), liver, gall bladder, kidneys, thyroid gland.
- Immune-compromised.
- Use of immunosuppressive drugs.
- Drug abuse, and not willing/able to stop this during the study.
- Continuous consumption of probiotics within 2 weeks prior to the study.
- Excessive alcohol usage (140 g/week, about 3.5 bottles/week or 4 glasses/day as soju).
- Participating in another clinical trial within 12 weeks prior to or during the study.
- Visiting a country affected by cholera in the past 1 month.

7.3 Recruitment

Subjects will be recruited using posters, advertisements (online and/or offline), etc. in Ewha Womans University Medical Center Mokdong Hospital and nearby area. Call center for recruitment will be available.

8. Dietary Guidelines

Subjects will be instructed to limit their diet during experimental period, as follows:

- Avoid the consumption of any product containing pre- or probiotics such as dietary fiber, oligosaccharides, etc. (Avoiding food list will be provided)
- Please limit intakes of any health functional foods
- Avoid alcohol consumption on all sampling days, as well as the day before sampling (day -1 and 0, day 13-17, day 27-31, day 41-43)

Subjects will also fill out recommended food score questionnaire at the beginning and ask to provide their diet record 3 days per week using smart phone app during the study.

9. Randomization and Blinding

As this will be a double-blind trial, the product will be blinded to the subject and blinded to the investigators and coordinators. The sponsor will provide coded samples for distribution to randomized subjects. The code list identifying the product consumed by each subject will be kept in an envelope with the sponsor and principal investigator, to be open only at the end of the study, after data analysis, or earlier in case of medical necessity.

10. Dosages and Administration

During the six-week trial, subjects will be randomly assigned to consume test or placebo products, one tablet per time point, for a total of two tablets a day (morning and evening). The study products will be dispensed in bottles. Subjects will be instructed to keep the product in the bottles and to pour out only as much as they need (one tablet, twice a day). Subjects are to return the unused study product (still in bottles), to the study site at the end of each period. Study team performs pill counts to assess study compliance and keep the returned product for return to the sponsor at the end of the study.

11. Study supplies

The study products will be manufactured by SEMPIO Foods Company. The test and placebo products will be identical in appearance and shape; there will be no difference to the naked eye, and the difference in weight will be minute. The labeling of the study products for this human study will be as follows:

1. The mark "For human experimental study only"<br>2. The code name of the product<br>3. Use-by date or best before date<br>4. Storage recommendation<br>5. The name of sponsor<br>6. Lot. No.<br>8. Unique code: Recorded according to randomization list<br>9. Directions for consumption

The sponsor will provide labeled product. The investigators will sequentially assign enrolled subjects to their respective randomization code list which will be prepared by the independent statistician. The research coordinator will confirm by signed written receipt the quantity of the study product supplied by the sponsor.

12. Outcome Measures

Local inflammatory response

- Fecal Calprotectin level as marker of gut inflammation

Change in cholera toxin-specific IgA and IgG antibody level

- In plasma, levels of cholera toxin-specific IgA and IgG

Local and systemic inflammatory responses

- β-defensins in feces
- I-FABP in plasma
- Blood cellular markers (leucocytes, lymphocytes, neutrophils, etc.)
- Cytokines/chemokines (IP-10, etc.)
- Acute-phase proteins (hsCRP, IL-1ra, etc.)

Gut microbiome

- Fecal microbiome

Others

- Bristol stool scale, etc

※ Biomarkers will be analyzed in Ewha Womans University Medical Center Mokdong Hospital, Department of Nutritional Science and Food Management in Ewha Womans University, NIZO food research and Seoul Clinical Laboratories.

※ Analysis for Omics can proceed if necessary.

13. Safety Outcome Variables

Safety and tolerability will be assessed throughout the study by measuring change from baseline (first measurement taken prior to supplementation) in each of the following: blood chemistry (CBC, AST and ALT), vital signs, body weight, and subject-reported adverse events.

14. Safety Procedures

Principal investigator will evaluate all adverse reports on a regular basis.

15. Adverse Event Monitoring

Adverse event or unusual signs will be monitored during the study. An adverse event is any unintended change in pathology or in anatomic, metabolic or physiologic functioning associated with the use of a study product, whether or not it is considered related. These changes are typically reflected by physical signs, reported symptoms, or laboratory data. Changes associated with normal activities not varying in frequency or magnitude from that ordinarily anticipated clinically are not considered adverse events.
Principal investigator will evaluate all adverse events as to their severity and relation to study product, to record the outcome and action taken, and to determine if they are serious. Adverse events will be graded by the principal investigator following the Common Terminology Criteria for Adverse Events v3.0 [CTCAE] criteria.
Principal investigator in accordance with good clinical practice will perform appropriate therapeutic action and follow-up measures. These actions and measures will continue until the condition is resolved and/or the etiology is identified.

Should the principal investigators become aware of a serious adverse event occurring or exacerbating in a subject at any time during the trial or within 90 days of receiving the last dose of study product, the event will be reported promptly (within 24 hours) by telephone to SEMPIO Foods Company and Biofood CRO. Co., Ltd. Serious adverse event will be also reported to IRB. Principal investigator will also need to judge the likelihood that the event was related to the study product and document this on the appropriate CRF.

Unknown.

Unrelated, which means that the study product did not cause the event.
Probably unrelated, which means that in all likelihood the study product did not cause the event.

Possibly related, which means that there is a reasonable possibility that the study product caused the event.

Related, which means that in all likelihood the study product caused the event.

16. Statistical analysis

Data analyses will be performed with intention-to-treat subject data (all data from subjects who have received the study product) or per-protocol subject data (only data from subjects who completed the study at least 80% compliant with study product). Variables will be tested for normal distribution and data transformation will be performed on skewed variables. Parametric or non-parametric analyses (dependent on outcome of the distribution of data) such as paired t-test, Student's *t*-test, Wilcoxon's rank sum test or Wilcoxon's signed rank test will be used to analyze the difference within each group or between the groups for continuous variables. Chi-square or Fisher's exact test will be used to analyze the difference between the groups for categorical variables. A mixed-effects model with group and time as fixed effects and subject as random effect will be applied to compare the changes between groups over the intervention period. The correlation between variables will be analyzed by Pearson's correlation and regression analysis will be performed. The data will be were analyzed using the SAS 9.3 statistical software. The difference between the groups will be tested two-sided for all study outcomes. P-values <0.05 are considered statistically significant.

17. Data Quality Assurance/Study Monitoring

Steps taken to assure the reliability and accuracy of the study data include the selection of qualified PIs, research coordinators, research assistants, and contract laboratory; review of protocol procedures with the PIs, protocol writer, and associated personnel prior to the study; and the presence of a research coordinator during the study days. The research coordinator will review case report forms for accuracy and completeness. The sponsor will carry out periodic on-site monitoring of study procedures and documents.

18. Compliance Monitoring

Subject interview and counting of study product returned to the clinic biweekly will be used to evaluate compliance. Non-compliance will be defined as consumption of <80% of the scheduled intakes of study product. Subjects will be called as appropriate to encourage compliance.

19. Ethics and Regulatory Considerations

After careful consideration of the relevant literature concerning the cholera vaccine and *Lb plantarum* K8 lysate being utilized and considering that they have been marketed for several years with no reported significant adverse events, we estimate there is a low level of risk involved in participation in this study. This study will be conducted according to Good Clinical Practice and the Declaration of Helsinki (2013). A signed written informed consent for the study will be obtained from all subjects before protocol-specific procedures are carried out. Subjects will be informed of their right to withdraw from the study at any time.

19.1 Institutional Review Board

An Institutional Review Board (IRB) must approve the clinical study protocol and consent form prior to study initiation. The IRB will be informed of all subsequent protocol amendments and of serious and unexpected adverse events occurring during the study, which are likely to affect the safety of the subjects or the conduct of the trial.

19.2 Informed Consent

The study will be explained verbally and the subjects will be given ample opportunity to inquire about details of the study. The subjects will also be given ample opportunity to read and understand the consent form before signing it. Consent will be documented by dated signature of the subject.

19.3 Subject Confidentiality

The investigators and research coordinators are responsible for ensuring that subjects' confidentiality will be maintained. Samples will be stored in a coded manner. The link between study code and subject identification information will be stored at a locked place. We do not destroy the files that can relate the ID to the study code. Case report forms or other documents will identify subjects by a code, and not by name. The research coordinator will keep a subject enrollment log showing codes. At the end of the study all identifiers will be stripped from all study related documents.

19.4 Withdrawal of Subjects from the Study

Subjects may be removed from the study for the following reason:

- A subject requests discontinuation;
  the principal investigator initiates removal for medical or compliance reasons;
- Occurrence of an adverse event that is considered serious by the principal investigator;
- Occurrence of an illness that affects the subject's further participation;
- Any other reason considered by the principal investigator to be necessary for a subject's safety;
- Non-compliance with study procedures.

In the event that a subject is withdrawn from the study the reason for the withdrawal will be documented in the case report form.

19.5 Subject Compensation

Compensation of 40,000 won will be paid to the subject at the time of each visit from visit 1 (day 0) to visit 7 (day 43). In case of drop-out, the compensation will be paid only for the completed visit. In addition, compensation for online survey (40,000 won) will be paid on visit 7 (day 43) only to the subject who completes the online questionnaire (Bristol stool scale).

20. Records

20.1 Deviations from Protocol

All changes to the protocol must be documented by amendments to the protocol signed by the principal investigators and the sponsor. If the amendment represents a substantial change to the protocol, it (and a revised informed consent form) will be submitted for approval to the IRB.

20.2 Case Report Forms

All data generated by the methods described in the study protocol will be recorded on the case report forms.

20.3 Record Retention

Ewha Womans University Medical Center Mokdong Hospital will retain the Consents and Case Report Forms in a secure location for three years after the date of release of an integrated report, or longer if so required by law.

21. Termination of Study

Ewha Womans University Medical Center Mokdong Hospital reserves the right to terminate the study at any time. In terminating the study, Ewha Womans University Medical Center Mokdong Hospital will assure that adequate consideration is given to the protection of the subjects' interest.

22. Management of human materials

The blood and feces collected in this study will be coded with IDs combining the screening number and the visit number, and then stored in the freezers for human materials in Ewha Womans University Medical Center Mokdong Hospital and Department of Nutritional Science and Food Management of Ewha Womans University. And it will be managed by the human materials management ledger. The person responsible for the management and storage of the

human materials shall be the principal investigator and the access right shall be allowed to research coordinator. It will be used for the analysis of functional and safety biomarkers according to the protocol.

The matters concerning the disposal and secondary use will depend on the period of preservation of the human materials, availability for secondary use, the inclusion of personally identifiable information which would be selected by the subjects in the consent form for human materials. Secondary use means the use of human materials for comprehensive research purposes. Even if human materials are used for secondary use, it shall be anonymized and managed so that personal information cannot be identified. Also, the collected human materials will be stored, moved and discarded in accordance with the standards and methods in accordance with Article 13 of the Waste Control Act, immediately after the end of the human materials preservation period selected by the subject. If the research is terminated abnormally, it will be disposed of in accordance with the procedure set out in the Enforcement Decree of the Bioethics and Safety Act.

23. References

1. EFSA guideline for gut function

2. A consideration of biomarkers to be used for evaluation of inflammation in human nutritional studies. Br J Nutr 109, S1-S34 2013

3. Dietary factors and low-grade inflammation in relation to overweight and obesity. Br J Nutr 106, S1-S78 2011

4. Geier, M. S., Butler, R. N., Giffard, P. M., and Howarth, G. S. (2007) *Lactobacillus fermentum* BR11, a potential new probiotic, alleviates symptoms of colitis induced by dextran sulfate sodium (DSS) in rats. *Int. J. Food Microbiol.* **114(3),** 267-274.

5. Mileti, E., Matteoli, G., Iliev, I. D., and Rescigno. M., (2009) Comparison of the immunomodulatory properties of three probiotic strains of Lactobacilli using complex culture systems: prediction for in vivo efficacy. *PLoS One.* **4(9),** e7056.

6. Chen, Y. P., Hsiao, P. J. Hong, W. S. Dai, T. Y., and Chen, M. J. (2012) *Lactobacillus kefiranofaciens* M1 isolated from milk kefir grains ameliorates experimental colitis in vitro and in vivo. *J. Dairy. Sci.* **95(1)**, 63-74.

7. Fujiki, T., Hirose, Y., Yamamoto, Y., and Murosaki, S. (2012) Enhanced immunomodulatory activity and stability in simulated digestive juices of *Lactobacillus plantarum* L-137 by heat treatment. *Biosci. Biotechnol. Biochem.* **76(5),** 918-922.

8. Zhou, Y. K., Qin, H. L., Zhang, M., Shen, T. Y., Chen, H. Q., Ma, Y. L., Chu, Z. X., Zhang, P., and Liu, Z. H. (2012) Effects of *Lactobacillus plantarum* on gut barrier function in experimental obstructive jaundice. *World J. Gastroenterol.* **18(30),** 3977-3991.

9. Kataria, J., Li, N., Wynn, J. L., and Neu, J. (2009) Probiotic microbes: do they need to be alive to be beneficial? *Nutr. Rev.* **67(9),** 546-550.

10. Adams, C. A. (2010) The probiotic paradox: live and dead cells are biological response modifiers. *Nutr Res Rev* **23(1),** 37-46.

11. Zakostelska, Z., Kverka, M., Klimesova, K., Rossmann, P., Mrazek, J., Kopecny, J., Hornova, M., Srutkova, D., Hudcovic, T., Ridl, J., and Tlaskalova-Hogenova, H. (2011) Lysate of probiotic *Lactobacillus casei* DN-114 001 ameliorates colitis by strengthening the gut barrier function and changing the gut microenvironment. *PLoS One.* **6(11),** e27961

12. Ryu, Y. H., Baik, J. E., Yang, J. S., Kang, S. S., Im, J., Yun, C. H., Kim, D. W., Lee, K., Chung, D. K., Ju, H. R., and Han, S. H. (2009) Differential immunostimulatory effects of Gram-positive bacteria due to their lipoteichoic acids. *Int. Immunopharmacol.* **9(1),** 127-133

13. Kang, S. S., Ryu, Y. H., Baik, J. E., Yun, C. H., Lee, K., Chung, D. K., and Han, S. H. (2011) Lipoteichoic acid from *Lactobacillus plantarum* induces nitric oxide production in the presence of interferron-gamma in murine macrophages. *Mol. Immunol.* **48(15-16),** 2170-2177.

14. Kim, H. G., Lee, S. Y., Kim, N. R., Lee, H. Y., Ko, M. Y., Jung, B. J., Kim, C. M., Lee, J. M., Park, J. H., Han, S. H., and Chung, D. K. (2011) *Lactobacillus plantarum* lipoteichoic acid down-regulated Shigella flexneri peptidoglycan-induced inflammation. *Mol. Immunol.* **48(4),** 382-391.

15. Ahn, Y. S., Park, M. Y., Shin, J. H., Kim, J. Y., Kwon, O. (2014) Lysate of Probiotic Lactobacillus plantarum K8 modulate the mucosal inflammatory system in dextran sulfate sodium-induced

colitic rats. *Kor J Food Sci Ani Res* **34**:829-835.
