# Supplementary material for: Study protocol for cholera vaccination as a model to measure the inflammatory response in the gut: A case of modulation with a Lactobacillus plantarum K8 lysate
Source: PLoS One. 2023 Feb 21;18(2):e0281817. doi: 10.1371/journal.pone.0281817 (PMC9942990; doi:10.1371/journal.pone.0281817)
Supplement: Attachment — Submitted filename: Response to Letter (PONE-D-22-04882).doc [file pone.0281817.s003.doc]

**Response to Letter**

Manuscript ID: PONE-D-22-04882

Title: Study protocol for cholera vaccination as a model to measure the inflammatory response in the gut: A case of modulation with a *Lactobacillus plantarum* K8 lysate

Dear Editor,

Thank you very much for your consideration of our manuscript. We respond to your queries as below.

**Comment**

1. Thank you for your response to our recent queries. Further to this, we note the the document you have provided is also dated March 3rd 2022. Could you please provide a copy of the original (signed or stamped) dated IRB/research ethics committee approval letter as an 'Other' file.

🡺 The date of the document is the date of issuance of the English version of IRB/research ethics committee approval letter. We have attached the original IRB/research ethics committee approval letter written in Korean.

2. Could you please also comment on why the principal investigator listed on your ethical approval document provided (Hee Jung, Choi) is not listed as an author on the current submission?

🡺 Professor Hee Jung Choi contributed to recruiting and managing participants. She joined after the protocol was developed. However, since clinical trials were conducted at the hospital, Professor Choi was designated as the principal investigator according to Korean regulations. Nevertheless, she provided her perspective as a doctor, we add Professor Choi as the author.
