# Supplementary material for: Study protocol for cholera vaccination as a model to measure the inflammatory response in the gut: A case of modulation with a Lactobacillus plantarum K8 lysate
Source: PLoS One. 2023 Feb 21;18(2):e0281817. doi: 10.1371/journal.pone.0281817 (PMC9942990; doi:10.1371/journal.pone.0281817)
Supplement: Attachment — Submitted filename: Response to Reviewers (PONE-D-22-04882).doc [file pone.0281817.s004.doc]

**Response to Reviewers**

Manuscript ID: PONE-D-22-04882

Title: Study protocol for cholera vaccination as a model to measure the inflammatory response in the gut: A case of modulation with a *Lactobacillus plantarum* K8 lysate

Dear Editor,

Thank you very much for your consideration of our manuscript and request for a revised version. We have copied and pasted all reviewers’ comments and addressed each one individually. As you will see, we made a number of changes in our manuscript to incorporate the questions and suggestions by the reviewers as thoroughly as possible. Below is a list of the changes following the order of the comments.

**Reviewer #1**

1. Why was the study population made up of only male individuals?

🡺 We thank the reviewer for this important comment.

The regulation of immune responses differs due to the presence of different hormones in males and females. The menstrual cycle represents hormonal fluctuations for immune function and regulation. In particular, the number of immune cells and regulated activity during the four-week cycle, as demonstrated in the case of regulatory T cells.

Recently, several studies have demonstrated relevant gender differences in microbiota composition that can account for differences in peripheral immunity between genders as well as intestinal immunity. In the research conducted on rodents, female mice showed increased microbial diversity compared to male mice.

Hence, the reproductive condition of females that may be related to microbiota results as confounder factors should be considered. This is the reason for the participants of this study being made up of only males **(Lines 270-278)**.

2. What did the authors consider healthy bowel habits?

🡺 We conducted a questionnaire on bowel habits. This study confirmed healthy bowel habits as a person who defecates regularly at least four times a week and once a day (less than two times).

We added in **Lines 133-134**.

3. Why was the 6 week supplementation time with probiotics chosen? What is the justification for this period?

🡺 It was intended to improve immunity by taking probiotics for 2 weeks before the first vaccination, taking them for 2 weeks between the first vaccination and the second vaccination, and taking them for 2 weeks after the second vaccination to demonstrate the effect of the probiotics with vaccine administration.

4. Why did the authors not assess fecal secretory IgA?

🡺 Oral cholera vaccinations are used to induce an immune response in the intestine to prevent cholera infection. However, oral vaccines can also affect the immune response of other mucosal tissues. Oral vaccination primarily interacts with the immune system through the oral cavity to the small intestine and induces the expression of vaccine-specific IgA in gastrointestinal and tissue-specific homing of B cells and T cells. The aim of the measurement of cholera toxin-specific IgA was to evaluate whether oral cholera vaccination is able to induce potent IgA responses in serum. We added **Lines 283-289**.

5. What about serum LPS and zonulin levels?

🡺 Fecal calprotectin level, β-defensin, I-FABP (Intestinal fatty acid-binding protein), and other cytokines/chemokines were biomarkers of this study protocol. This is important to understand the kinetics and the persistence of the effect of the vaccination on these markers.

6. Evaluating the variations in the intestinal microbiota would be of great value to the study.

🡺 We thank the reviewer for this important comment. We are planning to measure the microbiome after breaking the blind. The study is ongoing. We also have included the sentences in **Lines 263-265**.

7. Improve the conclusions? What is the clinical significance of the study? What diseases can be studied with this model?

🡺 This paper is for clinical protocol. Clinical analysis is currently in progress. We will analyze and publish the paper afterward.
